# Miniaturization in Biocatalysis

**DOI:** 10.3390/ijms11030858

**Published:** 2010-03-02

**Authors:** Pedro Fernandes

**Affiliations:** IBB–Institute for Biotechnology and Bioengineering, Centre for Biological and Chemical Engineering, Instituto Superior Técnico, Av. Rovisco Pais, 1049-001, Lisboa, Portugal; E-Mail: pedro.fernandes@ist.utl.pt; Tel.: +351-218-419-189; Fax: + 351-218-419-189

**Keywords:** multi-well plates, microreactors, enzyme immobilization, flow reactors, fluidic properties

## Abstract

The use of biocatalysts for the production of both consumer goods and building blocks for chemical synthesis is consistently gaining relevance. A significant contribution for recent advances towards further implementation of enzymes and whole cells is related to the developments in miniature reactor technology and insights into flow behavior. Due to the high level of parallelization and reduced requirements of chemicals, intensive screening of biocatalysts and process variables has become more feasible and reproducibility of the bioconversion processes has been substantially improved. The present work aims to provide an overview of the applications of miniaturized reactors in bioconversion processes, considering multi-well plates and microfluidic devices, update information on the engineering characterization of the hardware used, and present perspective developments in this area of research.

## 1. Introduction

Biocatalysis relies on the catalytic ability of enzymes to promote the chemical conversion of educts into given products. Biocatalysis hence differs from fermentation processes, where *de novo* synthesis of molecules is made from carbon and energy sources [1]. The wide plethora of biological agents used as sources for biocatalysts, combined with the use of different environments as bioconversion media and suitable engineering approaches for the characterization of the bioconversion process, allowed for an undeniable success when the implementation of biocatatytic systems at laboratory scale is considered. Enzyme technology is applied for the production of bulk and speciality chemicals; drugs and intermediates; goods for food and feed industry; soaps, cleaning and personal care products; textile, oil, wastewater and waste processing, occasionally aiming at incorporation in sustainable energy production processes; production of plastics and similar synthetics; biosensors. Enzyme technology is furthermore used in several medical, materials and bioremediation applications [2–5]. Despite of a comparatively lower start, the implementation of bioconversion processes at an industrial scale, meaning products commercialized at a scale over 100 kg on an annual basis, is almost doubling every decade. A recent study established the number of bioconversion processes at industrial scale at about 150 [1]. Alongside, the market for enzymes has increased in an almost exponential manner from the 1960s to 2000. Some shots in the arm can account for such a development pattern. Some of those significant milestones include the use of detergent proteases, the development of enzymatic methods for starch processing and the application of enzymes in synthesis. Alongside came also new approaches for the improvement or identification of more efficient biocatalysts. These include the introduction of recombinant DNA technology as well as new methodologies for assessing biodiversity (*viz*. metagenome approach, sequence-based discovery). The latter overcame the limitation of traditional screening methods, ascribed to the low number of microorganisms that can be cultured from those in a typical soil sample [6–8]. The introduction of recombinant DNA technology, and later, of enzyme engineering, through directed and particularly through random mutagenesis, namely using directed evolution, has actually strongly contributed to a revitalization of biocatalysis in the last 25 years [7,9–13]. This resurgence was particularly welcome, since it allowed both to further expand the potential of biocatalysis and to contribute for the rebuttal of some criticisms of biocatalysis, which were voiced in roughly the same time frame. Some key papers were also published, that suitably addressed such criticisms [11,14–16]. A common remark from these papers is the need to avoid generalizations.

One of the criticisms of biocatalytic systems that is still put forward is that they are relatively slow to implement [2,17]. The rapid and consistent emergence of microscale processing techniques that have been successfully implemented recently, based in the use of microwell plates and/or miniature/micro- bioreactors, are contributing much to speed up the development of bioconversion systems. Many of these developments take place alongside with research efforts within the same field focused in fermentation and cell culture systems [18–23]. The high level of parallelization achieved in such devices allows for the high throughput required in the different stages of development of a bioconversion process. These may include the biocatalyst screening step; the generation of libraries of recombinant biocatalysts with improved activity/selectivity/stability; the selection of suitable operational conditions, both for biocatalyst production or for performing the biocatalytic step, including strategies for enzyme immobilization [24–34]. Within the wide array of configurations and working volumes available, the present work will focus on miniature (less than 10 mL) bioreactors, multiwall plates and microchannel reactors. Alongside with the high number of variables and parameters that can be assessed simultaneously, miniaturization also allows for significant reductions in manpower, as well as in the amounts of reagents required and in waste production, with significant contributions to cost reduction in process development and well as making the process greener [35–37]. Miniaturization down to the microfluidic environment presents further advantages, namely a more rapid heat exchange and mass transfer, as compared to conventional, larger scale systems. Within the scope of process intensification, microscale processing techniques have also been developed that are dedicated to the downstream step [38], namely involving centrifugation [39], chromatography [40], liquid-liquid extraction [41,42] or microfiltration [43].

## 2. Miniature Systems

### 2.1. Multi-well Plates as Bioreactors

Multi-well plates have been intensively used for high throughput screening of enzyme activity, in assays based in the production (depletion) of a chromogenic or fluorogenic product (substrate), allowing for the simultaneous evaluation of up to 10^6^ different variants [29,44]. Screening can be performed in liquid media or in solid media [45]. Within this scope, multi-well plate based screening has been mostly used for profiling hydrolases, and, to a minor extent, proteases [46]. Multi-well plates have also been used to evaluate the kinetics of biotransformation systems [24,47]. Dynamic pH monitoring as strategy for the determination of kinetic parameters has also been performed, using as model system the hydrolysis of 4-nitrophenol catalyzed by penicillin acylase, and based in the color change of a pH indicator [48]. A similar approach was developed recently for assessing fat quality in foods [49]. The test relied on the influence of fat composition in lipase activity and was monitored by absorbance shift. Recently, Rachinskiy and co-workers developed an experimental set-up based in multi-well plates that allows for the high throughput of enzyme performance and stability [50]. Enzymes are submitted to extreme conditions for short time periods in order to anticipate their long term stability under milder conditions. The methodology combines the high level of parallelization provided by multi-well plates, thorough temperature control, on line techniques to monitor shifts of given parameters (*viz*. pH) along the time course of the reaction and software for processing the raw data.

The commercialization of multi-well plates with pH (or dissolved oxygen) sensitive sensor spots embedded in each well, coupled to on-line data acquisition (www.presens.de), favors the implementation of this approach for evaluation of kinetic data, although limited to bioconversion systems where pH (oxygen) changes are involved (Table 2).

To be representative such studies require an understanding of the impact in mass transfer phenomena and flow dynamics due to operation on a microliter scale. In the particular case of multi-well plates, mixing is typically promoted by shaking rather than by stirring. Some exceptions to this pattern are the use of micro stir bars in the screening of activity for the whole cell reduction of 6-bromo-β-tetralone to 6-bromo-β-tetralol within yeast and rhodococci libraries [53] and in the assessment of mixing conditions in the production of l-erythrulose promoted by a transketolase [47]; or gas sparging through porous membranes inserted into the base of each well, used for microbial cell growth [54]. The shape of the well (*viz*. round well *vs* square well), along with the static surface area to volume ratio and shaking intensity has been shown to affect biocatalyst activity in bioconversion systems requiring oxygen, hence gas-liquid mass transfer, such as the Baeyer-Villiger oxidation of bicyclo[3.2.0]hept-2-en-6-one [24]. Such behavior has been ascribed to the pattern of oxygen transfer. Given the relevance of gas-liquid transfer in oxygen dependent reactions, particular care has been given to the identification of operational condition that favor oxygen transfer into the liquid phase, hence preventing operating in biased conditions due to oxygen limitations. This is mostly due to: (a) enhanced shaking intensity within given boundaries, a lower boundary where oxygen transfer rate is distinguishable from static cultures, and an upper boundary, corresponding to a kinetic limited regime or to liquid spillage out of the vessel; (b) increased shaking amplitude; (c) a high static surface to volume ratio where it is assumed that oxygen transfer to the liquid phase mostly occurs at the surface of the liquid rather than by entrained bubbles; (d) improved mixing in square shaped wells, which emulate baffles [24,55–59]. Operation under suitable gas-liquid volumetric mass transfer coefficient, k_l_a, matching those observed in conventional laboratory (or higher)-scale bioreactor has been shown feasible in miniaturized systems, either based in given multi-well plates and in miniature bioreactors, and this parameter used as criterion for scale-up of fermentation systems [59,60–63]. When multi-well plates are used, a higher ratio of the gas–liquid exchange area to the volume of the bulk liquid can be made available. Miniaturization gives relevance to physical properties such as surface tension, which counters gravitational forces and bubbling, that are further enhanced alongside with size reduction. This pattern is therefore relatively unnoticed in 24-well plates, and increasingly noticeable in 48-, 96- and 384-well plates [55,64,65]. Given those two physical characteristics, a reasonable oxygen transfer rate can be obtained even under a poor degree of mixing of the bulk liquid in multiwall plates [65]. Optical methods for measuring surface tension of liquids contained in multiwall plates have hence been developed, in order to adequately cope with the high throughput nature of the process [66].

When gas-liquid transfer is not needed, the degree of mixing required is no longer related to a given demand for mass transfer between the two phases. Mixing takes place at different levels, macro-, meso- and micro-mixing. Macro-mixing relates to mixing at a macroscopic scale, hence at the scale of vessel, and leads to large-scale distribution patterns, such as residence-time distribution and concentration gradients. Meso-mixing relates to the blending of feed columns of fluid with the bulk fluid, and corresponds to the process where the reactant incoming into the reactor moves away from the plume and is reduced to turbulent eddies. Unlike macro-mixing, that relates to the whole lifetime of an element of fluid in the vessel, meso-mixing relates only to the early moments of an element of fluid that has entered the reactor. Micro-mixing promote contact of fluids at the microscopic or molecular scales. Micro-mixing comprehends a viscous-convective alteration of elements of fluid followed by diffusion [67,68]. In a relative timescale, micro-mixing and reaction times are comparable [69]. With miniaturization, micro-mixing tend to be predominant. Furthermore, and alongside with the decrease in linear dimensions, shorter mixing times are needed, hence diffusion limitation are minimized. Although this pattern is typical of microfluidic devices [20,85], the pattern is already observed at multi-well plate level. Thus, while assessing the relevance of mass transfer in miniaturized system, using as model system the production of l-erythrulose catalysed by transketolase, Matosevic and coworkers noticed that only when the 24-well reaction plate was vigorously mixed, the initial reaction rate statistically matched data obtained from non-shaking 96-well plate [47]. Multiwell plates are typically made of polypropylene or polystyrene, with the later being preferred due to the lack of leakage of toxic substances [56].

### 2.2. Microfluidic Systems

**Figure 1.**
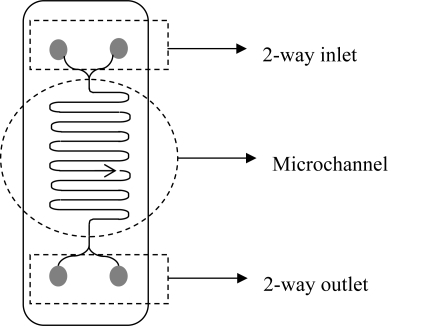
Scheme of a typical microfuidic device, with a microchannel and 2-ways inlet and outlet. Microreactors are commercially available from Chemtrix, CPC–Cellular Process Chemistry Systems GmbH Ehrfeld Mikrotechnik, Micronit/Future Chemistry, Microinnova, Mikroglas, Syrris, among others.

Microfluidic reactors provide another format for miniaturized bioconversion systems. Unlike micro-well plates, microfluidic reactors allow for processing small volumes (10^−9^ to 10^−12^ m^3^) of fluid within channels, where at least one dimension is smaller than 1 mm (typically tens to hundreds of micrometers), and are compatible with continuous flow mode of operation. Microfluidic devices themselves are concomitantly of small dimensions, from some mm to micrometers [20,70,73,85]. Microfluidic reactors consist of a network of miniaturized channels, embedded in a flat surface, comonly called “chip” [20,71,72] (Figure 1). Along with this chip type of microreactors, simpler microcapillary devices are also extensively used, where the microchannel is the reaction space, and thus no control of microfluidics is required. On the other hand, they do not allow for the integration of different processes into one reaction device, unlike chip microreactors [73].

Scaling-up a bioconversion process for conventional bioreactors encompasses the use of a suitable engineering criterion, that is to be maintained constant throughout scales, such as the volumetric oxygen transfer coefficient (k_l_a), volumetric power consumption, mixing time or impeller tip speed, when microreactors are considered the operational conditions can be scaled by simply operating multiple systems in parallel, a process termed numbering-up or scaling-out. This feature is particularly attractive since it takes away the technical and financial risks associated with scaling a given process in traditional manner.

#### 2.2.1. Materials

A wide array of materials has been used to build microfluidic reactors, among them polymers, glass, metals, and ceramics. Polymer-based devices typically present low cost and allow for the easy integration of flow control systems (viz. pneumatic valves, sensors), but are often sensitive to organic solvents [74]. Poly(dimethylsiloxane), or PDMS, a soft, optically transparent elastomer, is a typical choice for building microreactors [75]. This low-cost polymer is stable in aqueous environment and due to its low interfacial free energy there is a low probability of other polymers or fluids reacting with or attaching to its surface [75–77]. PDMS is however prone to swell or even dissolve in the presence of some organic solvents, and has a relatively limited temperature stability, although the later is compatible with common biological applications. Besides, its elastic properties lead to some restrictions in the design, since a high aspect ratio of the microchannels may result in pairing, where parallel structures attach to each other, whereas low aspect ratio can result in sagging, hence making the recommended aspect ratio for PDMS structures to be within 0.2 and 2 [76,78,79]. Other polymers commonly used are SU-8, an epoxy photosensitive epoxy resin that allows for adequate aspect ratio structures to be obtained [74,80], and silicone. The later polymer is actually also widely used for microreactor building. Not only fabrication methods that are well established for semi-conductor chip production are easily amenable, but also silicone has high thermal conductivity, allowing for excellent heat transfer capability. Furthermore, when oxidized, silicone becomes chemically inert towards most chemicals and solvents [71,74]. Glass is another widely used material for building microfluidic reactors, despite its rigid, brittle nature. Glass is however chemically inert to most reagents and solvents, and since it is transparent allows for visual inspection of events inside the reactor. Besides methods for fabrication, such as photolithography, are well established [70,71,78]. Stainless steel is the preferred metal for building microreactors. A well-established material, resistant to organic solvents and a wide array of chemicals, enables operation under pressure and high temperatures. Typically these reactors are larger than those made from polymers or glass, and are mostly used for pilot-plant or production scale, although they are also available for academic research purposes [70,71]. Methods for fabrication of microfluidic chip reactors include laser ablation, micro-injection molding, surface treatment and photolithography, perhaps the most popular. A detailed description of the different methodologies can be found in [70].

#### 2.2.2. Flow in Microfluidic Devices

**Figure 2.**
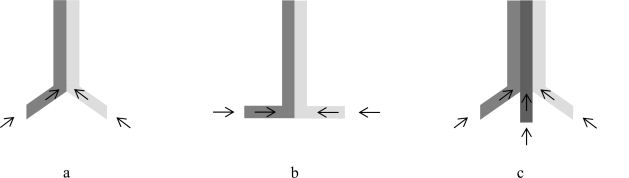
Examples of junctions in microfluidic devices: (a) Y junction; (b) T junction (c) Ψ junction. Arrows suggest direction of flow.

Continuous flow is by large the preferred mode of operation in large-scale processing in the chemical industry [81], so the use of microfluidic devices brings high-throughput, lab-scale process optimization closer to the large-scale processes [82–84]. Flow in microfluidic reactors can be single phase or multiphase, where in the later case two or more phases are separately added to the reactor, typically in co-current mode. Most common junctions for multiphase flow are T, cross, interdigitated multilamellar mixer, Ψ and Y junctions, the latter being widely used in two-phase bioconversion systems [70,85] (Figure 2).

The flow dynamics and mass transfer in a microfluidic environment, however, differs from larger systems. Given the small dimensions, the flow regime is typically laminar, interfacial forces are dominant over gravitational forces and since turbulence is not present due to the high viscous forces, mixing relies on molecular diffusion [85,86]. The laminar flow favors the control and modeling of the reaction, and provide high surface area to volume ratio (>200) and interfacial areas, a feature particularly advantageous in two-liquid extractive bioconversion systems [20,73,87]. In such environment Reynolds and Bond numbers are usually small and the Peclét number is high [88,89]. The Reynolds number, Re, represents the ratio inertial to viscous force (1):

(1) $$\text{Re}=\frac{\text{u}\;\text{L}}{\nu }$$

where u is the linear velocity of the fluid, L is the hydraulic diameter of the microchannel and ν is the kinematic viscosity. The Bond number, Bo, expresses the ratio of gravitational to surface tension forces (2):

(2) $$\text{Bo}=\frac{\Delta \rho \;\text{g}\;{\text{L}}^{2}}{\gamma }$$

where Δρ and γ are the density difference and the interfacial tension between two phases, respectively, and g is the gravitational acceleration. The Peclét number, Pe, expresses the ratio of forced convection to diffusion (3):

(3) $$\text{Pe}=\frac{\text{u}\;\text{L}}{\text{D}}$$

where D is the diffusion coefficient. Also relevant in the characterization of multiphase flow is the Weber number, We, which provides the ratio between inertial and surface tension forces, (4), and is useful in the analysis of thin film flows and formation of droplets and bubbles. Despite of the small paths involved, We in the range of some hundreds can be obtained in microfluidic devices [70]

(4) $$\text{We}=\frac{\rho \;\text{L}\;{\text{u}}^{2}}{\gamma }$$

Finally, the capillary number, Ca, (5), is also a relevant dimensionless parameter in the analysis of multiphase flow. It relates viscous (elongation) to surface tension forces acting across the interface:

(5) $$\text{Ca}=\frac{\mu \;\text{u}}{\gamma }$$

where μ is the viscosity of the fluid. Tipically much lower than 1 [89], Ca allows to distinguish two-phase flow patterns and the mechanisms of break-up in T-junctions [90].

**Figure 3.**
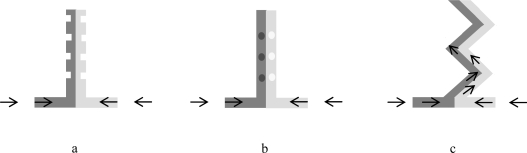
Examples of typical configurations to enhance mixing in microchannels: (a) obstacles on the wall of the microchannel; (b) obstacles in the microchannel (c) zig-zag microchannel. Arrows suggest direction of flow.

A common approach to improve diffusion-induced mixing is to use long and narrow microchannels (high aspect ratio channels) [86,91]. When channel dimensions are of tens of microns, the diffusion path is small enough for the mixture of different fluid streams to be complete in a few seconds, a time span that is considerably enlarged, to tens of seconds, when the channel dimensions are in the hundreds of microns [92]. Micro-mixers have hence been developed as to increase the interfacial surface and therefore decrease significantly the diffusion length [73,92]. Micro-mixers can be of passive or active nature. In the former case, mixing is promoted by diffusion or chaotic advection, whereas in the later, the flow is disturbed by the action of an external source. Passive mixing can be obtained by lamination, where the inlet streams are divided into several substreams, and then combined together in a single stream; or by chaotic advection, induced by special geometries in the mixing channel, such as zig-zag microchannel, and obstacles on the wall or in the microchannel [73,93] (Figure 3).

Typically fluids are delivered to the microreactor through the use of either syringe or HPLC pumps, within a broad range of flow rates, from μL min^−1^ to L min^−1^ [71]. The connection between microreactors and macroscopic fluid delivering device is performed using a large array of components, such as Luer-to-Luer or Luer-to-barb connectors, common HPLC fittings and high-pressure interconnect devices to interface with standard capillary tubing [94].

#### 2.2.3. Applications of Microreactors in Biocatalysis

The use of microreactors for the analysis and development of bioconversion systems has mostly relied in the use of enzymes or at most cell lysates as biocatalysts [73]. Irrespective of the approach used, the microreactor approach has systematically been shown to outperform conventional batch-wise operation, namely when the rate of substrate processing is considered, which could be somehow anticipated, given the favorable mass and heat transfer characteristics of the former [73,95,96]. Different configurations have been used within the scope of microreactors. The simplest form is the operation in a single liquid phase, where substrate and enzyme aqueous solution are fed separately to the microreactor and the reaction takes place along the microchannel. This was used for the hydrolysis/transgalactosylation by glycosidase [97], for the bioluminescent reaction between ATP and luciferin, promoted by luciferase to ultimately yield oxyluciferin and AMP [98], and for the oxidation of 3,4-dihydroxy-l-phenylalanine (l-DOPA) with laccase [95]. In all cases the efficiency of the approach was demonstrated, which somehow paved the way for more complex approaches. One of these is a likely extension of the former, since organic-aqueous or ionic liquid-organic solvent two-liquid phase systems were used. This allowed for the conversion of sparingly water-soluble substrates or for the enzymatic resolution of chiral compounds. The experimental set-up for these occasionally featured Ψ-shaped microreactors, allowing for three independent feeding channels. Further details are given in Table 3.

Enzymes can be advantageously used in immobilized form since this strategy allows for increased volumetric productivity and often improves stability. Continuous mode of operation is made possible, mostly using a packed-bed reactor (PBR) configuration, since this overcomes the continuous stirred tank reactor (CSTR) configuration, by allowing for higher conversion or space-time yields [2,104,105]. In order to retain the biocatalyst, particularly if in free form, membrane reactors operating in CSTR mode are commonly employed, where it is assumed that the contents of the reactor are perfectly mixed [2]. Other screening devices can be used if the biocatalyst is attached to a carrier or encapsulated. *Ex-situ* filtration or centrifugation, followed by recycling back into the reactor, is an alternative strategy for enabling continuous mode of operation [105]. Optional to PBR configuration, is the expanded or fluidized bed reactor, where the enzyme particles are retained by a hydrodynamic balance between gravity and drag forces promoted by the up-flow substrate stream [105,106]. The approaches commonly used for immobilization in conventional multiphase biocatalysis can also be used in microreactors, with particular focus on covalent methods, cross linked enzyme aggregates (CLEA) and adsorption (specific and non-specific) methods [107]. The experimental set-ups are preferably either chip-type reactors with activated channel walls where the enzyme binds, or enzyme immobilized monolith reactors, where a support is packed inside a capillary tube (Table 4).

#### 2.2.4. Visualization and Quantification of Fluid Flow

Gaining the in-depth in fluid mechanics, mixing and heat transfer required for the engineering characterization of miniature and microreactors has required both hardware and software tools, able to collect the required data, process them and generate models representative of the system dynamics. Contribution to the visualization of fluid flow has been made possible through the use of adequate high speed cameras, often combined with (micro) particle image velocimetry, (μ)PIV, and software for image analysis [17,121–127]. Generation of detailed predictive models, representative of the dynamics of flow, which can incorporate reaction data, has been made possible with computational fluid dynamics (CFD). CFD allows therefore for the design and analysis of a given system, as well as for the simulation of fluid flow, (bio)chemical reaction, mass and heat transfer [19,62,87,128,129]. The models developed within the scope of CFD methods are typically anchored in the Navier–Stokes equations and in the convection-diffusion equation [19,130].

## 3. Conclusions

The introduction of miniaturized devices in biocatalysis is becoming widespread and is clearly contributing to speed up the rate of process development in a cost effective manner. The large parallelization capability allows for simultaneously evaluating a large array of possibilities, hence improving process optimization. Miniaturization encompass the use of multi-well plates and microfluidic devices, each playing preferred roles in the development, characterization and ultimately implementation of bioconversion processes to production scale. The use of microfluidic reactors is the mainstay of this later feature, since processes are easily scaled by parallelization of devices, ruling out the need for elaborate and not always fully reproducible, scale-up procedures. Furthermore, when compared to typical bioreactors, microreactors present the advantage of high surface-to-volume ratio, high heat and mass transfer rates and ease of maintenance. The level of development and the effective application of this concept to bioconversion systems strongly relies on its multidisciplinary character, combining biotechnology, (micro)electronics and (micro)mechanics, microscopy and software for image analysis and data processing. Microreactors are becoming increasingly effective with the concomitant miniaturization of monitoring and controlled fluid delivery devices. Furthermore, the commercial availability of microreactors is increasing, which clearly favors the dissemination of te concept. Given the development trends in the field, and aiming not to be pictured as a panacea for the (bio)chemical (as biocatalysis should also not be) it can be expected that the relevance of miniaturization within biocatalysis and biotransformations is to further increase, and alongside, to further establish this area as a key player within the scope of a sustainable development.

## Figures and Tables

**Table 1.** Advantages, criticisms and comments on biocatalysis.

**Advantages**	**Criticisms**	**Comments**
High selectivity of enzymes (substrate, stereo-, region- and functional group selectivity	Narrow range of substrates for a given enzyme	The remarkable chemical selectivity of enzymes favors production of single stereoisomers, minimizes side reactions, eases downstream and reduces pollution. Although some enzymes seem limited to a single substrate (*viz* catalase) many others, namely hydrolases, act on a wide range of substrates
Operation under mild conditions	Enzymes are limited to aqueous environments	The ability to act as catalyst at atmospheric pressure and relatively low temperatures (as compared to chemical catalysts) decreases production costs Several enzymes are active in non-aqueous environments, and occasionally present novel activities under such media.
Environmentally friendly	Enzymes only accept low substrate loadings.	As proteins, enzyme catalysts are fully biodegradable, and present no relevant hazard for humans (but for occasional allergic reactions), unlike most chemical catalysts. Biocatalysis has low energy demands, hence minimizing emissions of greenhouse gases Although in nature enzymes are typically faced with low titers, they have been shown to perform efficiently under high substrate concentrations
High catalytic efficiency	Enzymes are too expensive	High turn-over numbers, If the cost issue is addressed on a price per kg basis of an enzyme and a transition metal this remark is not obvious, far from it. Yet, and although enzymes are major players in some area, such as detergents, where low cost is a major asset, their use in the production of plenty of commodities as well as in the energy sector (viz. biodiesel) is far from the choice in industry
Enzymes can be modified to enhance activity, selectivity, stability	High sensitivity of enzymes and operation in a limited range of pH and temperature	Although low stability of enzymes is often claimed, many enzymes display high operational stability. Enzyme immobilization has partly contributed to this, as well as to widen the mode of operation, albeit at an increase in production costs. Adequate processing of the exhausted immobilized biocatalyst may bring along further costs

**Table 2.** Characterization of bioconversion systems using multi-well plates.

**Microreactor**	**Bioconversion system**	**Comments**	**Ref.**
96-well plate	Hydrolysis of 4-nitrophenyl acetate to 4-nitrophenol and acetic acid catalyzed by free penicillin acylase	Evaluation of kinetic parameters. Design of experiments based on the color change of pH indicator along the time course of the reaction	[48]
24-round and 96-round and 96-square deep well plates	Baeyer-Villiger oxidation of bicyclo[3.2.0]hept-2en-6-one to (-)-(1S,5R)-2-oxabicyclo[3.3.0]oct-6-ene-3-one and (-)-(1S,5R)-3-oxabicyclo[3.3.0]oct-6-ene-2-one catalyzed by free whole cells of *Escherichia coli* with cyclohexanone monooxygenase activity	Evaluation of operational parameters (viz. well shape, shaking frequency, biocatalyst concentration, filling volume) in the outcome of the bioconversion. Glycerol was used as source of reducing power for regeneration of the NADP^+^/NADPH system. Validation of the “sacrificial well” approach. Comparison of kinetics in multi-well plate and stirred reactor. Quantification of substrate/products by GC	[24]
24-square well plates	Sitosterol side-chain cleavage to 4-androstene-3,17-dione (AD) using whole resting cells of *Mycobacterium* sp. NRRL B-3805	Establishes the feasibility of microtiter plates as platforms for the characterization of multi-enzyme bioconversion systems and as tools for solvent selection in complex bioconversion systems. Highlights some key operational parameters that have to be considered (*viz*. evaporation, chemical interaction of solvent and plate material)	[30,51]
24-square and 96-round well plates	Production of L-erythrulose from lithium hydroxypyruvate and glycolaldehyde using *E. coli* lysates with transketolase activity	Evaluation of the statistical significance of initial reaction rate data at multi-well scale. Effect of mixing in the bioconversion pattern. Further validation of the “sacrificial well” approach. Quantification of substrate/products by HPLC	[47]
96-round well plates	Ester hydrolysis catalyzed by esterase	Establishes a multi-well platform for the fast characterization of biocatalysts. Relies on fluorescence techniques for on-line monitoring of the product formed. A mathematical model was developed, which allows for relating the pH-shift that takes place during the reaction, and the concentration of the resulting product.	[50]
96-well plates	Alcoholysis of p-nitrophenyl acetate with 1-propanol promoted by a esterase in anhydrous environment	Screening for suitable methodologies for enzyme immobilization in multi-well plates	[52]

**Table 3.** Examples of microfluidic systems for enzyme catalysis in liquid phase.

**Microreactor**	**Bioconversion system**	**Comments**	**Ref.**
Chip type microreactor, made of glass, with Y-junctions at the inlet and at the outlet, continuous mode of operation. Oxygen (half) saturated L-DOPA and laccase solutions fed from each inflow	Oxidation of L-DOPA with laccase in full aqueous media	High (roughly 90%) conversion yields were obtained for residence times under 2 minutes. Model predictions, based in the reaction-diffusion equation, provided a good approach to experimental data	[95]
Chip type microreactor, made of glass, with Y-junctions at the inlet and at the outlet, continuous mode of operation. n-Hexane and substrates; and buffered enzyme solution fed from each inflow. In the Y-shaped outlet buffer and n-heptane phases were recovered	Synthesis of isoamyl acetate in n-heptane/buffer catalyzed by lipase, using acetic acid as acyl donor	Faster reaction rates were observed in the microfluidic system, when compared to batch runs. Model simulations obtained by numerical solution of non-linear systems provided a good fit to experimental data	[96]
Chip type microreactor, made of Poly(methyl methacrylate), PMMA, with Y-junction at the inlet, continuous mode of operation	Hydrolysis of p-nitrophenyl-β-D-galactopyranoside and transgalactosylation on p-nitrophenyl-2-acetamide-2-deoxy-β-D-glucopyranoside, both promoted by galactosidase	Hydrolysis was performed in fully buffered media, whereas transgalactosyation was performed in buffer-acetonitrile solvent system, to minimize reverse reactions. Both reactions were enhanced as compared to the batch system	[97]
Chip type microreactors, made of PDMS, with Y-junction at the inlet, continuous mode of operation	Bioluminiscent reaction promoted by luciferase	The reaction was performed in full aqueous media, with luciferin/luciferase and ATP solutions fed to each side of the junction. The microfluidic technique allowed for the determination of Michaelis-Menten rate constants with a single experiment	[98]
Chip type microreactor, made of glass, with Y- or Ψ-junction at the inlet, and Y- and single junction at the outlet, respectively, continuous mode of operation. The Ψ-junction was used for the separate inflow of ionic liquid (IL), enzyme and isoamyl alcohol; IL acetic anhydride and enzyme; and n-heptane	Synthesis of isoamyl acetate in n-heptane/1-butyl-3-methylpyridinium dicyanamide, catalyzed by lipase, and with acetic anhydride as acyl donor	Lipase was retained in the interface given its amphiphilic nature. The system allowed for simultaneous esterification and product recovery, showed a 3-fold increase in reaction rate when compared to conventional batch runs, and higher productivity. Parallel or slug flow could be observed depending on the relative flow rate of the ionic liquid and of the organic solvent	[99]
Chip type microreactor, made of glass, with Y-junctions at the inlet and at the outlet, continuous mode of operation. Aqueous phase with enzyme and KCN, and organic phase containing aldehyde were fed from each inflow	Synthesis of optically pure cyanohydrins using a cell lysate containing S-selective hydroxynitrile lyase	The crude cell lysate allowed for enantioselective synthesis of cyanohydrins in microchannels with a reaction rate and selectivity only achieved in larger batch mode under intense shaking, where a stable emulsion was formed. No clogging of the microchannels was observed	[100, 101]
Chip type microreactor, made of glass, with Y-junctions at the inlet and a single outlet, continuous mode of operation. Aqueous phase containing the enzyme and n-decane containing substrates were fed from each inflow	Esterification of propionic acid and n-butanol catalyzed by lipase	Kinetic parameters obtained in microfluidic system matched those obtained in conventional batch mode of operation. Activation and inactivation patterns were also similar in both scales	[102]
Chip type microreactor, made of glass, with Y-junctions at the inlet and a single outlet, continuous mode of operation. Aqueous phase containing the enzyme and iso-octane containing substrates were fed from each inflow	Dehalogenation of p-chlorophenol catalyzed by laccase	The surface of the microchannel was partially modified with octadecylsilane groups to provide a hydrophobic nature, and thus phase separation at the outlet of the microchannel	[103]

**Table 4.** Examples of microfluidic systems with immobilized enzymes.

**Microreactor**	**Bioconversion system**	**Comments**	**Ref.**
Capillary tubes with a frit at one end, packed with dried cross-linked (+)-γ-lactamase, mixed with controlled pore glass (120–200 mesh, 500-Å nominal pore diameter) in a 1:1 ratio	Conversion of benzamide to benzoic acid using γ-lactamase. Other substrates were screened, namely amides, with γ-lactam emerging as the preferred substrate, although the immobilized enzyme easily hydrolyzed several aromatic amides	The immobilized enzyme was stable for 6 h at 80 °C and kinetic constants were determined in the microreactor. Packing the capillary tubes with the cross-linked enzyme without controlled pore glass led to prohibitive back pressure levels	[108]
Same as above, or monolith microreactor. Immobilization in monoliths was achieved by binding to the surface epoxide groups	Conversion of *N*-benzoyl-l-phenylalanine into l-phenylalanine catalyzed by l-aminoacylase	CLEA capillary column reactor and monolith reactos allowed for 100% conversion at 20ºC and 40 °C respctively, well below the optimum temperatue of 85 °C	[109]
Capillary glass tube Novozym® 435	Conversion of 1-methyl-cyclohexene to 1-methyl-cyclohexene oxide and epoxidation of alkenes in the presence of hydrogen peroxide	Effective transfer of a batch process to a packed-bed flow reactor, allowing for a significant reduction in reaction time. Furthermore, the flow reactor allowed for hydrogen peroxide to be used over prolonged periods of time	[110]
Cross-linked enzyme aggregates, CLEA, immobilized onto the inner wall of poly(tetrafluoroethylene), PTFE, microtubes (0.25 mm inner diameter)	Hydrolysis of acetyl-d,l-phenylalanine catalyzed byimmobilized acylase	Successful implementation in a microreactor configuration of a methodology for preparing CLEA applicable to electronegative enzymes	[111]
Capillary glass microreactor containing a silica monolith. Glucose oxidase or choline oxidase were separately immobilized on the surface of the polyethylenimine (PEI) coated monolith	Conversion of d-glucose to d-gluconic acid catalyzed by glucose oxidase or conversion of choline to choline acetate catalyzed by choline oxidase	Simple method for preparation of monolith with controlled porosity, allowing for low pressure drop and avoiding mass transfer limitations. Enzyme immobilization was effective on the PEI-activated surface of the monolith, through interaction due to the electronegative and the electropositive nature of the former and the later. Kinetic constants were easily established since on-chip electrochemical detection allowed fast monitoring of enzyme kinetics	[112]
Chip type microreactor, made of PDMS, supplemented with pyrogenic silicic acid as a filler and simultaneously providing hydroxy groups for surface chemistry. Enzyme was covalently immobilized on silanised walls of the microchannels by coupling with glutardialdehyde	Hydrolysis of lactose catalyzed by β-glycosidase. Operated in continuous mode and in aqueous phase	The microstructured enzyme reactor was effectively tested in continuous production. A residence time above 33 minutes was required to achive a conversion yield of 100 mM substrate in excess of 60%. The system endured 5 days of continuous operation	[113]
Chip type microreactor, made of PDMS, with the enzyme entrapped in the PDMS crosslinked matrix	Hydrolysis of urea catalyzed by urease. Operated in continuous mode and in aqueous phase	Urea conversion significantly decreased for flow rate above 0.064 cm^3^ min^−1^ for and initial substrate concentration of 100 mM. Promising results were also referred for operation with glucoamylase in starch hydrolysis	[114]
Stainless steel plate with 34 linear channels. Full volume of the reactor of 25 microL. The walls of each channel were coated with a layer of □-aluminum oxide for covalent immobilization of the enzyme. The layer was derivatized with derivatized with (3-aminopropyl)triethoxysilane and the amino groups activated with glutardialdehyde	Transglucosylation reactions catalyzed by β-glucosidase. 2-Nitrophenyl-β-d-glucoside, oNPGlc, or cellobiose were used as donor substrates and glycerol as acceptor, to obtain β-glucosylglycerol in two stereochemical forms, 1-*O*-β-d-glucosyl-*rac*-glycerol and 2-*O*-β-d-glucosyl-*sn*-glycerol. Hydrolysis of lactose to galactose and glucose with β–glucosidase. In both cases, reactions were performed in aqueous environment and in continuous mode	Residence times were within 0.2 to 90 s. High yields of βGG, roughly of 60% and 80%, based on cellobiose and oNPGlc converted, respectively. Near exhaustion of substrate (80%), yields about 120 mM of βGG from the reaction of 250 mM cellobiose and 1 M glycerol. Determination of kinetic parameters for lactose hydrolysis. Sustained hydrolysis of lactose (100 mM) at 80 °C was observed for 4 days, corresponding to a space-time yield of 500 mg glucose mL^−1^h^−1^ at a stable conversion in excess of 70%.	[115, 116]
Microreactor composed of PTFE tubing (0.5 mm inner diameter) with enzyme covalently linked. Glutaraldehyde and paraformaldehyde were used as crosslinkers	Hydrolysis of *N*-glutaryl-l-phenylalanine p-nitroanilide catalyzed by α-chymotrypsin. Operated in continuous mode and in aqueous phase	Hydrolysis yield was kept at 90% and above for a substrate concentration of 1 mM, in a continuous flow (4 μL min^−1^) for some days	[117]
Chip type microreactor with microchannels in PDMS with enzyme-containing poly(ethylene glycol) (PEG) hydrogel microstructures fabricated in microfluidic channels	Hydrolysis of p-nitrophenylphosphate with alkaline phosphatase. Operated in continuous mode and in aqueous phase.	A pH sensitive fluorophore was incorporated in the hydrogel microstructures to allow for reaction through the variation of the emission intensity ratio with pH. The immobilization approach system was reported to be also effective when applied to the applied to urea hydrolysis by urease.	[118]
Capillary poly(ether ether ketone) (PEEK) tubes, with inner diameters within 0.1–2.0 mm, filled with silica monolith-entrapped enzyme, produced by sol-gel methodology, from tetramethoxysilane and methyltrimethoxysilane	Transesterification between (*S*)-(-)-glycidol and vinyl *n*-butyrate catalyzed by a protease. Continuous operation in organic environment	The microreactor outperformed the batch reactor used for control regarding conversion, when operating at higher flow rates (from the total range of 4.0 × 10^−4^ to 5.0 mLmin^−1^). No changes in conversion were observed at a given superficial liquid velocity with variations in tube diameter. Moreover, the conversion increased with a decrease in the enzyme content. The whole suggested mass transfer limitations	[119]
Chip type microreactor, made of PDMS as a microfuidic fuel cell. Three enzymes were immobilized alongside the bottom wall of the single stream channel. Bilirubin oxidase (BOD)-adsorbed O2 cathode and a glucose anode prepared by co-immobilization of glucose dehydrogenase (GDH), diaphorase (Dp) and vitamin K3-modified poly-L-lysine, VK3-PLL.	Oxygen reduction catalyzed by BOD; Reduction of VK3/oxidation of NAD+ catalyzed by Dp and NADH regeneration catalyzed by GDH	The cell performance, based on output current, increased with channel height. However, the volume density of current and power were enhanced when cell height decreased	[120]
